# Simplified algorithm for reliability sensitivity analysis of structures: A spreadsheet implementation

**DOI:** 10.1371/journal.pone.0213199

**Published:** 2019-03-08

**Authors:** Mahdi Shadab Far, Hongwei Huang

**Affiliations:** Department of Geotechnical Engineering, Tongji University, Shanghai, China; Technische Universiteit Delft, NETHERLANDS

## Abstract

An important segment of the reliability-based optimization problems is to get access to the sensitivity derivatives. However, since the failure probability is not a closed-form function of the input variables, the derivatives are not explicitly computable and rather require a full reliability analysis which is computationally expensive. In this paper, a step-by-step algorithm has been presented to calculate the derivatives of the probability of failure and safety index with respect to the input parameters based on the advanced first-order second-moment (AFOSM) reliability method. The proposed algorithm is then implemented in a spreadsheet using Visual Basic for Application (VBA) programming language. Two geotechnical and structural examples are then presented to examine the program and describe the modeling procedure. The robustness of the proposed method is examined using a Gaussian random perturbation. The capability of the proposed method in the calculation of the sensitivity derivatives of the model uncertainty is explained in a separate section. Finally, the proposed model has been compared to the forward finite difference (FFD) method and the results are validated.

## 1 Introduction

Because of aleatory and/or epistemic uncertainties associated with loads and capacity of structures alike, deterministic models fail to provide a sufficiently reliable estimation of design variables [1]. In order to consider the uncertainties, probabilistic methods are thus preferred [2–4]. That is, reliability-based design optimization is adopted for structural design process [5]. More precisely, the philosophy of optimization tends to minimize the risk rather than focusing on the stress/strain level induced in structural elements. As such, the probability of failure should be limited to a certain extent [6, 7]. This can be achieved by considering the failure probability as a constraint in the optimization process. Mathematically speaking, this can be summarized as follows:

**Minimize**: the objective function, *f*(*b*, *μ*_*X*_)

**Subject to**: *P*_*j*_[*g*_*j*_(*b*, *X*) < 0] ≤ *P*_*Rj*_ and *j* = 1, 2, …, *m*

where *g*_*j*_(•) denotes the performance function (also known as limit state function (LSF)), *b* is the vector of deterministic design variables, *X* is the vector of random variables, and *P*_*Rj*_ is a certain level of failure probability to which the system is bound. One of the main steps in solving this optimization problem is to determine search direction based on function gradients, i.e. sensitivity analysis [8, 9]. Sensitivity analysis demonstrates change(s) in the quantity of response in terms of variations in design variable(s) [10–12]. This process is necessary since most of the available algorithms for solving the optimization problem need to access derivatives of failure probability with respect to input parameters [13]. A few algorithms have been proposed to tackle this issue. However, each has its own limitations. For instance, they are mostly based on the implicit algorithms which require cyclic calculations to estimate the derivatives; their performance is limited to specific distribution functions; can only cover the derivatives with respect to the mean or standard deviation of random variables; with any change in the performance function or random variable characteristics, their code should be thoroughly edited [14]. These limitations have made the structural reliability software packages dependent on utilizing classic methodologies such as finite difference method (FDM) for calculating sensitivity derivatives. However, FDM requires performing a complete reliability analysis at each step of the analysis to detect the effect of variation in input parameters on the failure probability. Therefore, a direct analytical method that can calculate the derivatives of failure probability with respect to input parameters is preferred.

It should be noted that the results of sensitivity analysis cannot be calculated explicitly in terms of design variables since the probability of failure is a non-classical function of design variables and random parameters [15]. In this paper, in order to overcome this problem, a simple algorithm is presented based on advanced first-order second-moment (AFOSM) reliability method, which determines the sensitivity formulae using only the first-order derivatives of performance function. To establish the proposed method, a general form of the problem is described and the model is formulated, followed by describing the process performed to calculate the failure probability and safety index with respect to target variables (i.e. mean and standard deviation of random variables). The described procedure is then presented as a step-by-step algorithm. Then, using the Visual Basic for Application (VBA) programming language, the proposed algorithm is implemented in a spreadsheet in Microsoft Excel environment [16]. To discuss the purpose of using the spreadsheet in this study, it should be mentioned that, when the number of random variables involved in the LSF function increases, the number of partial derivatives required for sensitivity analyses increases too, making the analysis complicated and time-consuming. However, the spreadsheet allows users to perform all the computations only by entering the variables and LSF function into the spreadsheet without engaging with any additional complexity. The algorithm described in this article can also be implemented in other programming languages. However, in that case, by any change in the performance function or input variables, the code should be edited as well. Thus, the spreadsheet environment seems to be more user-friendly in this case and therefore, is preferred to other programming languages. To examine the program and explain the modeling process, two examples including a geotechnical case study about the damage induced in a single-hole rock explosion and a structural problem about the fatigue crack growth under cyclic loading are presented and solved by the spreadsheet. The results are then compared and validated by forward finite difference (FFD) method.

## 2 Formulation of problem

Consider a system with a performance function *G*(*b*, *x*) where *b* is the vector of design variables and *x* is the vector of random variables. When AFOSM is employed for reliability analysis, the probability of failure is calculated as follows [17]:

$$P_{f}=1-\Phi \left(\beta \right),$$ (1)

where *β* is safety index and Φ(•) is cumulative density function of standard normal distribution. As such, the sensitivity of failure probability with respect to design variable (∂*P*_*f*_/∂*b*) can be defined as follows:

$$\frac{\partial P_{f}}{\partial b}=-\phi \left(\beta \right)\frac{\partial \beta }{\partial b},$$ (2)

where ∂*β*/∂*b* is the derivative of safety index with respect to design variable (*b*) and *ϕ*(•) is density function of standard normal distribution. The type of distribution function governing the variables is one of the important issues in the estimation of sensitivity derivatives. To address this issue in the proposed algorithm, the input variables are projected to the normal standard space using the change of variable. Then, the variables are entered into the algorithm as a vector of equivalent normal variables. This technique enables the algorithm to cover a wide range of probabilistic distribution functions. For this purpose, regardless of the type of distribution function governing the involved random variables, the standard normal form of variables (*U*), which is commonly used in reliability analysis, is calculated as follows [18]:

$$U=\frac{x-\mu_{x}}{\sigma_{x}}={\left\{U_{1},U_{2},\ldots ,U_{n}\right\}}^{T},$$ (3)

where *μ*_*x*_ and *σ*_*x*_ are the mean and standard deviation of random variables, respectively. With this assumption, the performance function can be rewritten based on standard normal variables in the form of *G*(*b*, *U*). Kwak and Lee [19] showed that, as a general case, the derivative of *β* with respect to design variable (*b*) can be expressed as follows:

$$\frac{\partial \beta }{\partial b}=\lambda \frac{\partial G}{\partial U}\frac{\partial U}{\partial b},$$ (4)

where λ, the Lagrange multiplier, can be calculated as follows:

$$\lambda =-\frac{1}{\left|\partial G/\partial U\right|}.$$ (5)

Now, two cases are considered here:

1. The design variable is the mean of random variables.
2. The design variable is the standard deviation of random variables.

These two scenarios are separately examined in the next sub-sections.

### 2.1 Reliability sensitivity with respect to mean

Taking the mean of random variables as the design variable, *b*, Eq (4) can be rewritten as follows:

$$\frac{\partial \beta }{\partial \mu_{x}}=-\frac{1}{\left|\partial G/\partial U\right|}\frac{\partial G}{\partial U}\frac{\partial U}{\partial \mu_{x}}.$$ (6)

The derivative ∂*U*/∂*μ*_*x*_ can be calculated from Eq (3):

$$\frac{\partial U}{\partial \mu_{x}}=\frac{\partial }{\partial \mu_{x}}\left(\frac{x-\mu_{x}}{\sigma_{x}}\right)=-\frac{1}{\sigma_{x}}.$$ (7)

Employing the chain rule, the derivative ∂*G*/∂*U* can be expanded as follows:

$$\frac{\partial G}{\partial U}=\frac{\partial G}{\partial x}\frac{\partial x}{\partial U}=\frac{\partial G}{\partial x}\sigma_{x}.$$ (8)

Substituting Eqs (7) and (8) into Eq (6), ∂*β*/∂*μ*_*x*_ can be simplified as follows:

$$\frac{\partial \beta }{\partial \mu_{x}}=-\frac{\frac{\partial G}{\partial x}\sigma_{x}}{|\frac{\partial G}{\partial x}\sigma_{x}|}\left(-\frac{1}{\sigma_{x}}\right)=\frac{\frac{\partial G}{\partial x}}{|\frac{\partial G}{\partial x}\sigma_{x}|}.$$ (9)

### 2.2 Reliability sensitivity with respect to standard deviation

In this section, taking standard deviation of random variables as design variable, *b*, Eq (4) is rewritten as follows:

$$\frac{\partial \beta }{\partial \sigma_{x}}=-\frac{1}{|\frac{\partial G}{\partial U}|}\frac{\partial G}{\partial U}\frac{\partial U}{\partial \sigma_{x}}.$$ (10)

Using Eq (3), the derivative ∂*U*/∂*σ*_*x*_ is calculated as follows:

$$\frac{\partial U}{\partial \sigma_{x}}=-\frac{\partial }{\partial \sigma_{x}}\left(\frac{x-\mu_{x}}{\sigma_{x}}\right)=-\frac{x-\mu_{x}}{\sigma_{x}^{2}}.$$ (11)

Substituting Eqs (8) and (11) into Eq (10), ∂*β*/∂*σ*_*x*_ is obtained as follows:

$$\frac{\partial \beta }{\partial \sigma_{x}}=-\frac{\frac{\partial G}{\partial x}\sigma_{x}}{|\frac{\partial G}{\partial x}\sigma_{x}|}\times \left(-\frac{x-\mu_{x}}{\sigma_{x}^{2}}\right)=\frac{\frac{\partial G}{\partial x}}{|\frac{\partial G}{\partial x}\sigma_{x}|}\frac{x-\mu_{x}}{\sigma_{x}}.$$ (12)

To further continue the calculations, a safety index should be defined. By definition, safety index is the shortest distance from the origin to the failure surface. Coordinates of the point on failure surface at the shortest distance to origin can be calculated as follows:

$$U^{*}=\beta \text{cos}\left(\theta_{u}\right),$$ (13)

where cos(*θ*_*u*_) (or cos(*θ*_*x*_)) represents the direction cosine of the unit outward normal vector (also known as sensitivity vector (*α*_*x*_)) and is calculated as follows [20, 21]:

$$\text{cos}\left(\theta_{x}\right)=\alpha_{x}=-\frac{\frac{\partial G}{\partial x}\sigma_{x}}{|\frac{\partial G}{\partial x}\sigma_{x}|}.$$ (14)

Substituting Eq (14) into Eq (13), U* is calculated as:

$$U^{*}=-\beta \frac{\frac{\partial G}{\partial x}\sigma_{x}}{|\frac{\partial G}{\partial x}\sigma_{x}|}.$$ (15)

Now, if the term (*x* − *μ*_*x*_)/*σ*_*x*_ in Eq (12) is replaced by U*, Eq (16) is obtained:

$$\frac{\partial \beta }{\partial \sigma_{x}}=\frac{\frac{\partial G}{\partial x}}{|\frac{\partial G}{\partial x}\sigma_{x}|}\times (-\beta \frac{\frac{\partial G}{\partial x}\sigma_{x}}{|\frac{\partial G}{\partial x}\sigma_{x}|})=\frac{{\left(\frac{\partial G}{\partial x}\right)}^{2}\sigma_{x}}{|\frac{\partial G}{\partial x}\sigma_{x}{|}^{2}}\beta .$$ (16)

### 2.3 Step-by-step algorithm

So far, the problem has been formulated and the necessary relationships to calculate the sensitivity derivatives have been developed. In this section, a step-by-step algorithm is presented to facilitate the process. To begin, it is required to identify input variables and determine their statistical characteristics, including means and standard deviations. In addition, a performance function should be determined as a function of involved variables. This part of modeling should be completed before the main calculation. Furthermore, following the steps listed below, sensitivity derivatives are estimated.

Step 1 Derivatives of performance function with respect to involved variables are evaluated at the mean values.
Step 2 Performance function is evaluated at the mean value. Additionally, the standard deviation of performance function, *σ*_*G*_, is estimated as follows:
  $$\sigma_{G}=|\frac{\partial G}{\partial x}{|}_{\mu }\times \sigma_{x}|.$$ (17)
Step 3 Safety index, *β*, and probability of failure, *P*_*f*_, are calculated as follows:
  $$\beta =\frac{\mu_{G}}{\sigma_{G}},$$ (18)
  $$P_{f}=1-\Phi \left(\beta \right).$$ (19)
Step 4 Derivatives of safety index with respect to the mean of the involved variables, ∂*β*/∂*μ*_*x*_, are calculated according to Eq (9).
Step 5 Derivatives of failure probability with respect to the mean of the involved variables, ∂*P*_*f*_/∂*μ*_*x*_, are estimated according to Eq (2).
Step 6 Derivatives of safety index with respect to the standard deviation of the involved variables, ∂*β*/∂*σ*_*x*_, are calculated according to Eq (12).
Step 7 Derivatives of failure probability with respect to the standard deviation of the involved variables, ∂*P*_*f*_/∂*σ*_*x*_, are approximated according to Eq (2).

**Fig 1:**
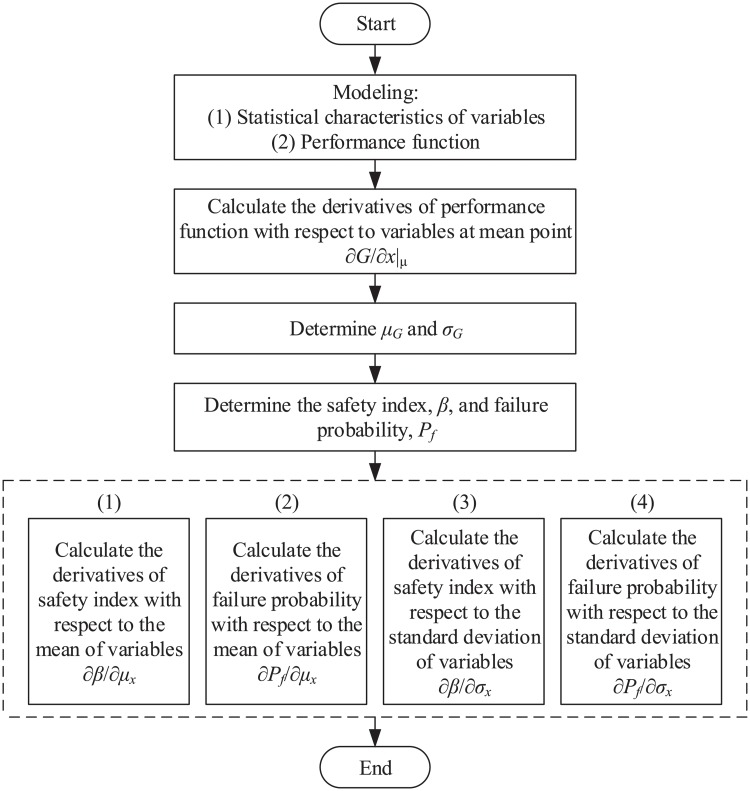
Schematic view of proposed algorithm.

The above steps are schematically presented in Fig 1.

## 3 Numerical example

A numerical example is presented herein to demonstrate the application of the described approach. The example is about a geotechnical project related to the damage induced in a single-hole rock explosion. Following the explosion, the resulting stress waves affect the surrounding environment severely, creating a highly damaged zone around the blast point, i.e. “crushed zone” [22, 23]. Many researchers have worked on the dimensional determination of the crushed zone. Djordjevic [24] presented the following equation to estimate the radius of the crushed zone:

$$r_{c}=\frac{r_{0}}{\sqrt{\frac{24T}{P_{b}}}},$$ (20)

where *r*_0_ is borehole radius (mm), *T* is tensile strength of the rock material (Pa), and *P*_*b*_ is borehole pressure (Pa). It is reported that *P*_*b*_ can be obtained as follows [25]:

$$P_{b}=\frac{1}{8}\rho_{0}D_{CJ}^{2},$$ (21)

where *ρ*_0_ is unexploded explosive density (kg/m^3^) and *D*_*CJ*_ is ideal detonation velocity (m/s). Substituting *P*_*b*_ from Eq (21) into Eq (20), *r*_*c*_ can be rewritten as:

$$r_{c}=\frac{r_{0}}{\sqrt{\frac{24T}{\frac{1}{8}\rho_{0}D_{CJ}^{2}}}}=r_{0}D_{CJ}\sqrt{\frac{\rho_{0}}{192T}}.$$ (22)

The input parameters in this model, such as detonation velocity or tensile strength of the rock mass could not be measured accurately and are always accompanied by a margin of uncertainties. Hence, substituting them into the model as fixed numbers leads to a deterministic estimation of the crushed zone radius. This is equivalent to assuming a crushed zone radius corresponding to 100% failure probability, which does not seem to be logical. A more rational solution towards the problem is to put the surety aside and define the parameters as random variables with certain means and standard deviations. This causes the output to be calculated as exceedance probability for the crushed zone radius. In general, defining the involved parameters as random variables and substituting them into the performance function, the model could turn from the deterministic to the probabilistic state, and could then be established as a reliability problem [26].

Suppose that the objective is to estimate the exceedance probability for a crushed zone radius of *r*_*c*_ = 400 mm. Thus, the performance function is defined as follows:

$$G=400-r_{0}D_{CJ}\sqrt{\frac{\rho_{0}}{192T}}.$$ (23)

Probabilistic characteristics of the involved variables are assumed as listed in Table 1. As seen, in addition to the distribution function governing the variables, corresponding means and standard deviations are also reported in this table. These parameters are required to calculate the equivalent normal variable vector as explained in Eq (3).

**Table 1:** Means and standard deviations of random variables.

Variable	Distribution function	Parameter 1	Parameter 2	Mean, *μ*	Standard deviation, *σ*
*r*_0_ (mm)	Lognormal [27]	4.316	0.363	80	30
*D*_*CJ*_ (m/s)	Lognormal [27]	8.506	0.149	5000	750
*ρ*_0_(kg/m^3^)	Normal [27]	950	200	950	200
*T* (MPa)	Weibull [27–29]	5.62	2.69	5	2

Now, adopting the algorithm described in the previous section, derivatives of safety index and failure probability with respect to the means and standard deviations of random variables are calculated.

Step 1 Derivative of performance function with respect to each random variable is calculated at the mean value.
  $$\frac{\partial G}{\partial r_{0}}{|}_{\mu }=-D_{CJ}\sqrt{\frac{\rho_{0}}{192T}}=-5000\times \sqrt{\frac{950}{192\times 5\times 10^{6}}}=-4.9739,$$ (24a)
  $$\frac{\partial G}{\partial D_{CJ}}{|}_{\mu }=-r_{0}\sqrt{\frac{\rho_{0}}{192T}}=-80\times \sqrt{\frac{950}{192\times 5\times 10^{6}}}=-0.0796,$$ (24b)
  $$\frac{\partial G}{\partial \rho_{0}}{|}_{\mu }=-\frac{1}{2}\frac{r_{0}D_{CJ}}{\sqrt{192T\rho_{0}}}=-\frac{1}{2}\times \frac{80\times 5000}{\sqrt{192\times 5\times 10^{6}\times 950}}=-0.2094,$$ (24c)
  $$\frac{\partial G}{\partial T}{|}_{\mu }=\frac{r_{0}D_{CJ}}{2}\sqrt{\frac{\rho_{0}}{192T^{3}}}=\frac{80\times 5000}{2}\sqrt{\frac{950}{192\times {\left(5\times 10^{6}\right)}^{3}}}=3.9791\times 10^{-5}.$$ (24d)
Step 2 Mean and standard deviation of performance function are approximated as follows:
  $$\mu_{G}=400-\mu_{r_{0}}\mu_{D_{CJ}}\sqrt{\frac{\mu_{\rho_{0}}}{192\mu_{T}}}=400-80\times 5000\times \sqrt{\frac{950}{192\times 5\times 10^{6}}}=2.0888,$$ (25)
  $$\begin{array}{cc} \sigma_{G} & =|\frac{\partial G}{\partial x}{|}_{\mu }\times \sigma_{x}|=({\left(\frac{\partial G}{\partial r_{0}}{|}_{\mu }\times \sigma_{r_{0}}\right)}^{2}+{\left(\frac{\partial G}{\partial D_{CJ}}{|}_{\mu }\times \sigma_{D_{CJ}}\right)}^{2}+{\left(\frac{\partial G}{\partial \rho_{0}}{|}_{\mu }\times \sigma_{\rho_{0}}\right)}^{2}\\ & +{\left(\frac{\partial G}{\partial T}{|}_{\mu }\times \sigma_{T}\right)}^{2}{)}^{\frac{1}{2}}=({\left(-4.9739\times 30\right)}^{2}+{\left(-0.0796\times 750\right)}^{2}+{\left(-0.2094\times 200\right)}^{2}\\ & +{\left(3.9791\times 10^{-5}\times 2\times 10^{6}\right)}^{2})=184.166. \end{array}$$ (26)
Step 3 Safety index and failure probability are estimated as follows:
  $$\beta =\frac{\mu_{G}}{\sigma_{G}}=\frac{2.0888}{184.166}=0.011342,$$ (27)
  $$P_{f}=1-\Phi (\beta )=1-\Phi (0.011342)=0.495475.$$ (28)
Step 4 Eq (9) is adopted to calculate derivatives of safety index with respect to the mean of variables.
  $$\frac{\partial \beta }{\partial \mu_{r_{0}}}{|}_{\mu }=\frac{\frac{\partial G}{\partial r_{0}}{|}_{\mu }}{\sigma_{G}}=\frac{-4.9739}{184.166}=-0.02701,$$ (29a)
  $$\frac{\partial \beta }{\partial \mu_{D_{CJ}}}{|}_{\mu }=\frac{\frac{\partial G}{\partial D_{CJ}}{|}_{\mu }}{\sigma_{G}}=\frac{-0.0796}{184.166}=-0.00043,$$ (29b)
  $$\frac{\partial \beta }{\partial \mu_{\rho_{0}}}{|}_{\mu }=\frac{\frac{\partial G}{\partial \rho_{0}}{|}_{\mu }}{\sigma_{G}}=\frac{-0.2094}{184.166}=-0.00114,$$ (29c)
  $$\frac{\partial \beta }{\partial \mu_{T}}{|}_{\mu }=\frac{\frac{\partial G}{\partial T}{|}_{\mu }}{\sigma_{G}}=\frac{3.98\times 10^{-5}}{184.166}=2.16\times 10^{-7}.$$ (29d)
Step 5 Eq (2) is employed to calculate derivatives of failure probability with respect to the mean of variables.
  $$\frac{\partial P_{f}}{\partial \mu_{r_{0}}}{|}_{\mu }=-\phi \left(\beta \right)\frac{\partial \beta }{\partial \mu_{r_{0}}}{|}_{\mu }=-\phi \left(0.011342\right)\times -0.02701=0.01077,$$ (30a)
  $$\frac{\partial P_{f}}{\partial \mu_{D_{CJ}}}{|}_{\mu }=-\phi \left(\beta \right)\frac{\partial \beta }{\partial \mu_{D_{CJ}}}{|}_{\mu }=-\phi \left(0.011342\right)\times -0.00043=0.00017,$$ (30b)
  $$\frac{\partial P_{f}}{\partial \mu_{\rho_{0}}}{|}_{\mu }=-\phi \left(\beta \right)\frac{\partial \beta }{\partial \mu_{\rho_{0}}}{|}_{\mu }=-\phi \left(0.011342\right)\times -0.00114=0.00045,$$ (30c)
  $$\frac{\partial P_{f}}{\partial \mu_{\rho_{0}}}{|}_{\mu }=-\phi \left(\beta \right)\frac{\partial \beta }{\partial \mu_{\rho_{0}}}{|}_{\mu }=-\phi \left(0.011342\right)\times 2.16\times 10^{-7}=-8.62\times 10^{-8}.$$ (30d)
Step 6 Derivatives of safety index with respect to the standard deviation of variables are calculated using Eq (12).
  $$\begin{array}{cc} \frac{\partial \beta }{\partial \sigma_{r_{0}}}{|}_{\mu } & =-\frac{{\left(\frac{\partial G}{\partial r_{0}}{|}_{\mu }\right)}^{2}\sigma_{r_{0}}}{\sigma_{G}^{2}}\times \beta =-\frac{{\left(-4.9739\right)}^{2}\times 30}{184.166^{2}}\times 0.011342\\ & =-0.0002482, \end{array}$$ (31a)
  $$\begin{array}{cc} \frac{\partial \beta }{\partial \sigma_{D_{CJ}}}{|}_{\mu } & =-\frac{{\left(\frac{\partial G}{\partial D_{CJ}}{|}_{\mu }\right)}^{2}\sigma_{D_{CJ}}}{\sigma_{G}^{2}}\times \beta =\frac{{\left(-0.0796\right)}^{2}\times 750}{184.166^{2}}\times 0.011342\\ & =-1.5891\times 10^{-6}, \end{array}$$ (31b)
  $$\begin{array}{cc} \frac{\partial \beta }{\partial \sigma_{\rho_{0}}}{|}_{\mu } & =-\frac{{\left(\frac{\partial G}{\partial \rho_{0}}{|}_{\mu }\right)}^{2}\sigma_{\rho_{0}}}{\sigma_{G}^{2}}\times \beta =\frac{{\left(-0.2094\right)}^{2}\times 200}{184.166^{2}}\times 0.011342\\ & =-2.9326\times 10^{-6}, \end{array}$$ (31c)
  $$\begin{array}{cc} \frac{\partial \beta }{\partial \sigma_{T}}{|}_{\mu } & =-\frac{{\left(\frac{\partial G}{\partial T}{|}_{\mu }\right)}^{2}\sigma_{T}}{\sigma_{G}^{2}}\times \beta =\frac{{\left(3.98\times 10^{-5}\right)}^{2}\times 2\times 10^{6}}{184.166^{2}}\times 0.011342\\ & =-1.0589\times 10^{-9}. \end{array}$$ (31d)
Step 7 Derivatives of failure probability with respect to the standard deviation of variables are estimated using Eq (2).
  $$\frac{\partial P_{f}}{\partial \sigma_{r_{0}}}{|}_{\mu }=-\phi \left(\beta \right)\frac{\partial \beta }{\partial \sigma_{r_{0}}}{|}_{\mu }=-\phi \left(0.011342\right)\times \left(-0.0002482\right)=9.9007\times 10^{-5},$$ (32a)
  $$\frac{\partial P_{f}}{\partial \sigma_{D_{CJ}}}{|}_{\mu }=-\phi \left(\beta \right)\frac{\partial \beta }{\partial \sigma_{D_{CJ}}}{|}_{\mu }=-\phi \left(0.011342\right)\times \left(-1.5891\times 10^{-6}\right)=6.3393\times 10^{-7},$$ (32b)
  $$\frac{\partial P_{f}}{\partial \sigma_{\rho_{0}}}{|}_{\mu }=-\phi \left(\beta \right)\frac{\partial \beta }{\partial \sigma_{\rho_{0}}}{|}_{\mu }=-\phi \left(0.011342\right)\times \left(-2.9326\times 10^{-6}\right)=1.1699\times 10^{-6},$$ (32c)
  $$\frac{\partial P_{f}}{\partial \sigma_{T}}{|}_{\mu }=-\phi \left(\beta \right)\frac{\partial \beta }{\partial \sigma_{T}}{|}_{\mu }=-\phi \left(0.011342\right)\times \left(-1.0589\times 10^{-9}\right)=4.2243\times 10^{-10}.$$ (32d)

## 4 Implementation in spreadsheet

Given that the process through which the reliability sensitivity derivatives were calculated was provided in the form of a step-by-step algorithm, it could be easily implemented. In this paper, Visual Basic for Application (VBA) was used to implement the proposed algorithm in Microsoft Excel spreadsheet for any given performance function (provided that it is differentiable) and any number of random variables. The designed spreadsheet and relevant codes are available along with the paper (or, can be downloaded from this link: https://sourceforge.net/projects/rsa-v1/). In the following, the modeling and analysis of the problem in the spreadsheet are described.

### 4.1 Describing the spreadsheet

**Fig 2:**
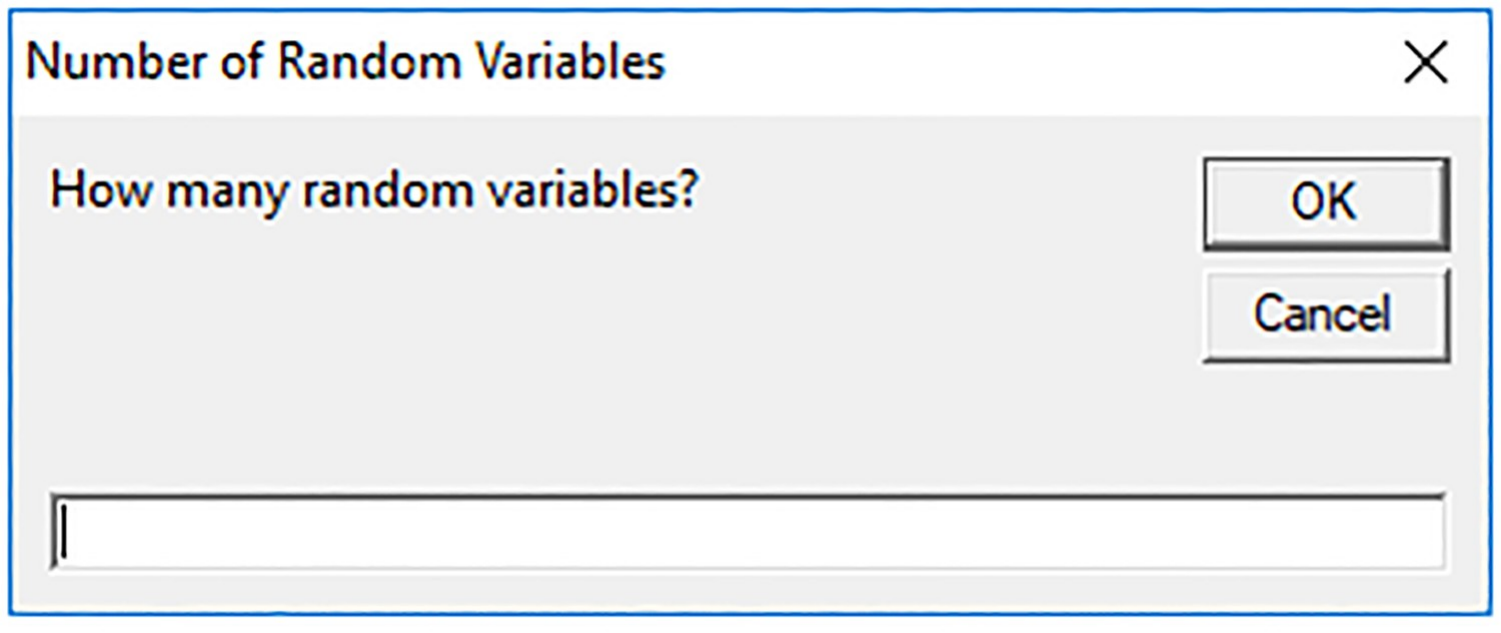
Window associated to the number of random variables.

Once the spreadsheet is opened, two buttons are seen at the top of the screen: “Modeling” and “Run Sensitivity”. It is important to note that, the macros should be enabled in Microsoft Excel to run the program. Usually, when a user opens a file containing macros, a yellow message bar appears at the top of the screen asking the user to enable the content. By clicking on “Enable Content,” it is possible to run macro codes. If this message is not available, the user can enable this feature by changing the macro security settings. To do it in Microsoft Office 2013, the user may follow the path: File≫Options≫Trust Center≫Trust Center Settings…≫Macro Settings, select the option “Enable all macros” and then mark the “Trust access to the VBA project object model.” To get started, the Modeling button is clicked. Then, as seen in Fig 2, a new window appears asking the user to input the number of random variables. For instance, there are four variables involved in the example presented in Section 3; thus, number 4 is inserted in this window and the OK button is clicked.

**Fig 3:**
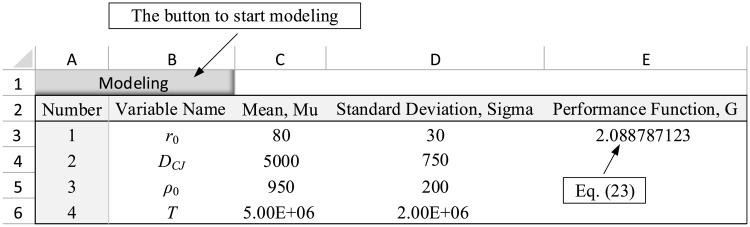
Data entry table in the spreadsheet for the example 1.

After defining the number of variables, a table is displayed in the spreadsheet to get the variables’ statistical information (Fig 3). One row is considered for each random variable in this table. In each row, column A shows the number of random variables (completed by default). Columns B, C, and D represent the name, mean, and standard deviation of the random variable, respectively, and must be completed in accordance with the statistical characteristics of random variables. After completing the information on this table, the performance function must be defined in cell E3 as a function of variables’ means using the Microsoft Excel formulae. By entering this function, the problem modeling is completed. With regard to the modeling section, It should be noted that:

- The performance function needs to be differentiable with its value defined at the mean point.
- There is no limitation applied on the number of variables involved in the problem. Any desired number of random variables can be modeled in the spreadsheet depending on the hardware limitations.
- Non-variable parameters with a fixed value can also be defined in the spreadsheet. In such cases, the parameter’s value must be defined as mean and standard deviation is set to zero.

To continue, simply click on the “Run Sensitivity” button to apply all of the calculations described in the previous section on the defined data. In the following section, two geotechnical and structural examples are solved in the spreadsheet and the results are compared with analytical calculations.

### 4.2 Solving examples by spreadsheet

#### 4.2.1 Example one: Rock blasting

**Fig 4:**
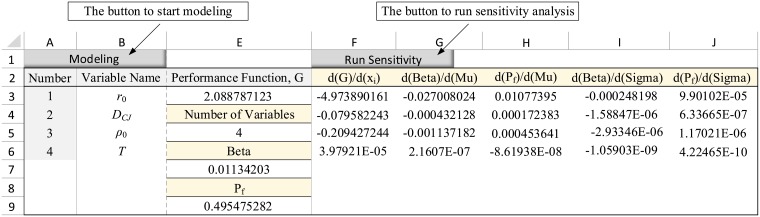
The output table in the spreadsheet for the example 1.

For the rock blasting example, as explained in Section 4.1, upon clicking on “Modeling” button and entering the number of variables as 4, a table is provided in the spreadsheet in which the characteristic of random variables are defined. Next, the performance function (Eq (23)) is defined in cell E3 as a function of variables’ means. In the presented numerical example, the function is: = 400-C3*C4*SQRT(C5/(192*C6)). The completed table is shown in Fig 3. Furthermore, by clicking on the “Run Sensitivity” button, the analysis was performed and the results were reported as a table in the spreadsheet (Fig 4).

In this table, the cell E5 displays the number of random variables. This value should be equal to the number of variables entered in the modeling step. The cell E7 represents the safety index, *β*. As seen, this cell equals to the value calculated via Eq (27). The cell E9 provides the failure probability as calculated via Eq (28).

The column G shows the derivatives of safety index, *β*, with respect to the means of random variables. Comparing the results listed in column G with the values calculated via Eqs (29a) to (29d), it is seen that the results are in good agreement. The column H provides derivatives of failure probability, *P*_*f*_, with respect to the means of random variables. This column corresponds to the values calculated via Eqs (30a) to (30d). Column I reports derivatives of the safety index, *β*, with respect to the standard deviation of random variables, closely approximating the results obtained in Eqs (31a) to (31d). Finally, column J presents the derivatives of failure probability, *P*_*f*_, with respect to the standard deviation of random variables, which are equivalent to the values calculated through Eqs (32a) to (32d).

#### 4.2.2 Example two: Fatigue crack growth

A new example concerning the growth of the fatigue crack in a finite rectangular plate with a corner crack under the cyclic stress is presented in this section. The classic solution to this problem could be represented by the *Paris* equation [30] as follows:

$$\frac{dl}{dN}=c\Delta K^{m},$$ (33)

where *m* = 3.32 is a constant coefficient, *l* is the crack length, *N* is the number of cycles, *c* is Paris constant, and Δ*K* is change in the stress intensity at the crack location. The stress intensity is calculated as follows:

$$K=\sigma \sqrt{\pi l},$$ (34)

where *σ* is the stress at the crack location. Variation in the stress intensity could be calculated as follows:

$$\Delta K=\Delta \sigma \sqrt{\pi l},$$ (35)

where Δ*σ* denotes the stress variation at the crack location. Substituting Δ*K* from Eq (35) into Eq (33) and sorting the equation with respect to *dN*, the following equation is obtained:

$$dN=\frac{dl}{c\Delta K^{3.32}}=\frac{dl}{c{\left(\Delta \sigma \sqrt{\pi l}\right)}^{3.32}}.$$ (36)

In order to estimate the number of cycles corresponding to the failure (*N*_*f*_), Eq (36) is integrated with respect to *l* from the initial dimension of the crack (*l*_*i*_) to the final dimension of the crack (*l*_*f*_):

$$N_{f}=\int_{l_{i}}^{l_{f}}\frac{dl}{c{\left(\Delta \sigma \sqrt{\pi l}\right)}^{3.32}},$$ (37)

where the final dimension of the crack (*l*_*f*_) is calculated as follows:

$$l_{f}=\frac{1}{\pi }{\left(\frac{K_{IC}}{1.1215\Delta \sigma }\right)}^{2},$$ (38)

where *K*_*IC*_ is the fracture toughness. Calculating the integral presented in Eq (37), *N*_*f*_ is estimated as follows:

$$N_{f}=\frac{l_{f}^{-0.66}-l_{i}^{-0.66}}{-0.66c{\left(1.1215\Delta \sigma \right)}^{0.32}\pi^{1.66}}.$$ (39)

The LSF function is now calculated as the exceeding of *N*_*f*_ from a limit state value of *N*_*all*_ = 4750 as follows:

$$\text{LSF}=4750-\frac{l_{f}^{-0.66}-l_{i}^{-0.66}}{-0.66c{\left(1.1215\Delta \sigma \right)}^{0.32}\pi^{1.66}}.$$ (40)

The probabilistic characteristics of the involved variables previously provided by operational experience [30] are given in Table 2.

**Table 2:** Probabilistic characteristics of the variables in example 2.

Random variable	Distribution type	Parameter 1	Parameter 2	Mean	Standard deviation
Δ*σ*	Lognormal	4.6	0.0998	100	10
*l*_*i*_	Lognormal	-4.71	0.472	0.01	0.005
*C*	Lognormal	-22.848	0.0997	1.2 × 10^−10^	1.2 × 10^−11^
*K*_*IC*_	Normal	60	6	60	6

**Fig 5:**
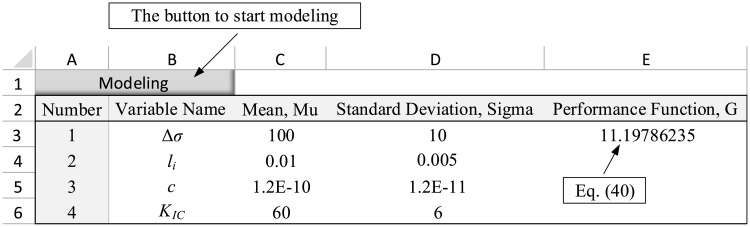
Data entry table in the spreadsheet for the example 2.

To define this problem in the spreadsheet environment, after clicking on “Model” option, the number of input variables is entered as 4. Then, the probabilistic characteristics of the variables are completed in accordance with Table 2. The result is shown in Fig 5.

**Fig 6:**
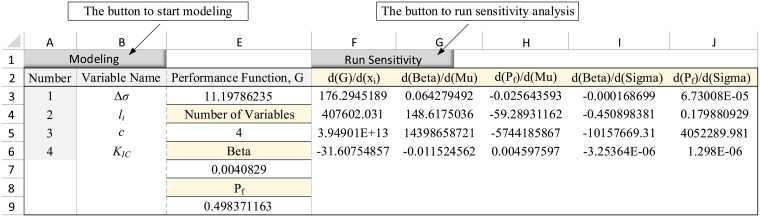
The output table in the spreadsheet for the example 2.

Next, the LSF function is defined in cell E3 in accordance with Eq (40). Then, by clicking on “Run Sensitivity” option, the sensitivity derivatives are calculated as shown in Fig 6.

As seen, the program given in the spreadsheet enables us to perform the sensitivity analysis by only knowing the statistical characteristics of the variables and the performance function, without any computational complexity.

## 5 Discussion

### 5.1 Robustness of the proposed algorithm

**Fig 7:**
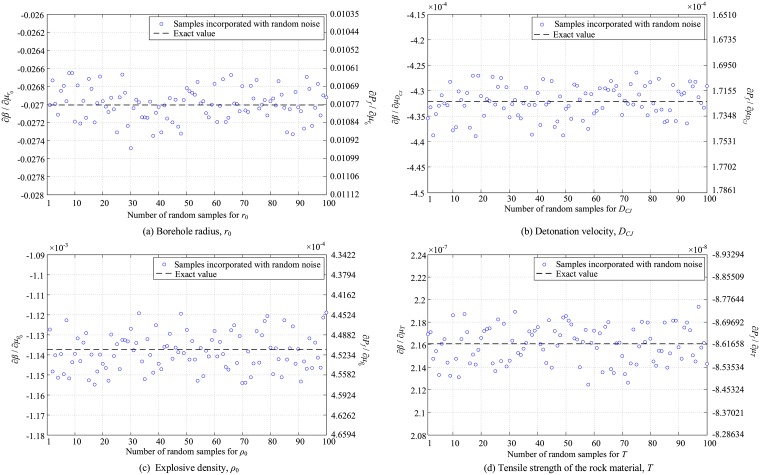
Response of the proposed algorithm to the random changes incorporated in (a) *r*_0_, (b) *D*_*CJ*_, (c) *ρ*_0_, and (d) *T*.

One of the important features of the algorithm presented in this paper is its robustness against changes made in the input variables. This is important because if there is difficulty in accurate modeling of the uncertainty, and the probabilistic parameters of the distribution functions governing the variables are accompanied by a margin of error, the algorithm could still provide a reliable answer. To numerically investigate this issue, a perturbation function was adopted to incorporate a random noise into the variables and measure the system response. For this purpose, a Gaussian distribution function with a coefficient of variation of 0.01 was utilized. The mean value of the Gaussian function was considered as the mean of random variables. One hundred random samples were generated for each random variable. The proposed algorithm was then used and the derivatives of the safety index and failure probability were calculated for each set of the random samples. The results are shown in Fig 7a–7d.

As seen, the algorithm showed a very robust response against the utilized changes presenting a close approximation to the actual result. In fact, the noises incorporated in the input variables have been accommodated by the algorithm and consequently, no significant changes were observed in the model output.

### 5.2 Model uncertainty

Another point that plays an important role in the probabilistic problems is the model uncertainty. A portion of the uncertainty is associated with the input variables that would be fed into the model by defining the probabilistic characteristics and probability distribution function of the variables. However, another type of uncertainty exists that is mostly related to the nature of the model and is the result of the model inability to accurately estimate the answer. In fact, this uncertainty originates from the difference between the estimated and exact output values. Thus, a new random variable, known as the model uncertainty variable, needs to be defined to address this type of uncertainty. Various algorithms have been proposed to describe how this variable should be incorporated in the problem formulation [26]. In the simplest case, a distribution function could be selected as the representative of the difference between the model output and the real data, and then added to the LSF function.

**Fig 8:**
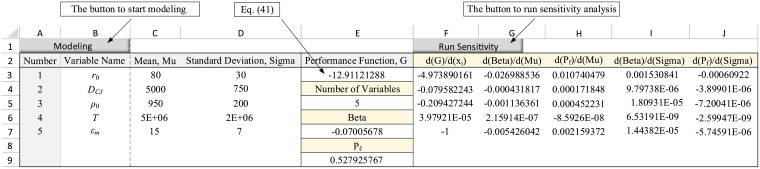
Sensitivity derivatives for the first example considering the model uncertainty.

This procedure is adopted in this section to address the model uncertainty. In the first example, assuming that the difference between the actual crushed zone radius and the estimated value from the Djordjevic model follows a normal distribution function with the mean *μ* = 15 and the standard deviation *σ* = 7, a new variable was added to the model. By considering the normal distribution function, we let the model uncertainty take both the positive and negative values because the estimated values can potentially be larger or smaller than the actual values. Hence, the LSF function is rewritten as follows:

$$G=400-(r_{0}D_{CJ}\sqrt{\frac{\rho_{0}}{192T}}+\varepsilon_{m}),$$ (41)

where *ε*_*m*_ represents the model uncertainty. Now, in order to calculate the sensitivity derivatives, the problem should be modeled with five random variables. The results in the spreadsheet environment are shown in the columns A to E of Fig 8. Next, by performing sensitivity analysis, the derivatives of the safety index and failure probability with respect to all the five variables were calculated. The results are shown in the columns F to J of Fig 8.

**Fig 9:**
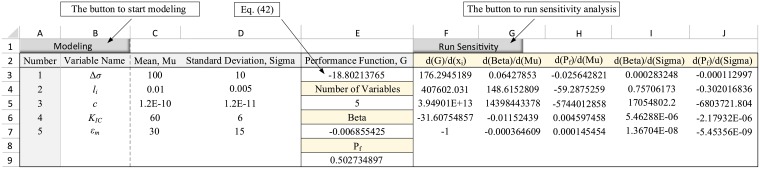
Sensitivity derivatives for the second example considering the model uncertainty.

The same calculations could be performed for the second example. For this purpose, the difference between the actual and estimated number of cycles leading to failure is assumed to follow a normal distribution function with a mean of *μ* = 30 and standard deviation *σ* = 15. Then, the limit state function is rewritten as follows:

$$\text{LSF}=4750-(\frac{l_{f}^{-0.66}-l_{i}^{-0.66}}{-0.66c{\left(1.1215\Delta \sigma \right)}^{3.32}\pi^{1.66}}+\varepsilon_{m}),$$ (42)

where *ε*_*m*_ denotes the model uncertainty. Utilizing the new LSF function, the five random variables were defined in the spreadsheet environment. The sensitivity derivatives were then calculated as shown in Fig 9.

As seen, the methodology presented in this paper could cover the model uncertainty and compute its sensitivity derivatives. As long as the uncertainty is modeled by a random variable in the performance function, the proposed method could be utilized to estimate the variability of failure probability and the reliability index associated with the target variable.

### 5.3 Validation

Finite difference sensitivity is adopted in this section to validate the algorithm provided in the spreadsheet. For this purpose, firstly, a small perturbation was applied on either mean or standard deviation while keeping the other variables constant. Then, the corresponding safety index and failure probability were computed using a reliability analysis. Next, forward finite difference method, as adopted in Eqs (43) and (44), was used to calculate sensitivities of safety index and failure probability.

$$\frac{\partial \beta }{\partial b}=\frac{\beta (b+\delta b)-\beta (b)}{\delta b},$$ (43)

$$\frac{\partial P_{f}}{\partial b}=\frac{P_{f}\left(b+\delta b\right)-P_{f}\left(b\right)}{\delta b},$$ (44)

where *δb* is the perturbation applied on parameter *b*. For instance, by creating one-percent perturbation in the mean of *r*_0_ in example 1, the mean value changed from 80 mm to 80.8 mm. Then, by keeping the other parameters constant, the program was run and new values of safety index and failure probability were calculated as: *β* = −0.01023 and *P*_*f*_ = 0.504081. Now, using Eqs (43) and (44), the sensitivity derivatives were calculated as follows:

$$\frac{\partial \beta }{\partial \mu_{r_{0}}}=\frac{\beta \left(\mu_{r_{0}}+\delta \mu_{r_{0}}\right)-\beta \left(\mu_{r_{0}}\right)}{\delta \mu_{r_{0}}}=\frac{-0.01023-0.01134203}{0.01\times 80}=-0.02696,$$ (45)

$$\frac{\partial P_{f}}{\partial \mu_{r_{0}}}=\frac{P_{f}\left(\mu_{r_{0}}+\delta \mu_{r_{0}}\right)-P_{f}\left(\mu_{r_{0}}\right)}{\delta \mu_{r_{0}}}=\frac{0.504081-0.495475}{0.01\times 80}=0.010757.$$ (46)

As observed, the values obtained from Eqs (45) and (46) were consistent with the cells G3 and H3 of Fig 8, respectively. In order to verify accuracy of the calculations for the rest of the variables, these calculations were repeated for all of the involved variables in examples 1 and 2. For this purpose, one-percent perturbation was applied to the mean and standard deviation of each variable separately before calculating their updated values. The results are presented in the form of 10 cases in Table 3.

**Table 3:** New means and standard deviations of random variables after applying perturbations.

Example	Case No.	Affected Variable	Perturbation in *μ*	Perturbation in *σ*	New Mean	New Standard Deviation
Example 1	Case 1	*r*_0_	0.01	0	80.8	30
	Case 2		0	0.01	80	30.3
	Case 3	*D*_*CJ*_	0.01	0	5050	750
	Case 4		0	0.01	5000	757.5
	Case 5	*ρ*_0_	0.01	0	959.5	200
	Case 6		0	0.01	950	202
	Case 7	*T*	0.01	0	5050000	2000000
	Case 8		0	0.01	5000000	2020000
	Case 9	*ε*_*m*_	0.01	0	15.15	7
	Case 10		0	0.01	15	7.07
Example 2	Case 1	Δ*σ*	0.01	0	101	10
	Case 2		0	0.01	100	10.1
	Case 3	*l*_*i*_	0.01	0	0.0101	0.005
	Case 4		0	0.01	0.01	0.00505
	Case 5	*c*	0.01	0	1.212 × 10^−10^	1.2 × 10^−11^
	Case 6		0	0.01	1.2 × 10^−10^	1.212 × 10^−11^
	Case 7	*K*_*IC*_	0.01	0	60.6	6
	Case 8		0	0.01	60	6.06
	Case 9	*ε*_*m*_	0.01	0	30.3	15
	Case 10		0	0.01	30	15.15

Then, considering the changes made on affected variable, the analysis was performed and the values of *β* and *P*_*f*_ for each of the ten cases in examples 1 and 2 were determined. The results are presented in Table 4.

**Table 4:** Safety index and failure probability for each of the ten cases.

Example	*r*_0_	*β*	Φ(*β*)	*P*_*f*_ = 1 − Φ(*β*)
Example 1	Case 1	-0.09133	0.46361	0.53639
	Case 2	-0.06960	0.47226	0.52774
	Case 3	-0.09084	0.46381	0.53619
	Case 4	-0.06998	0.47210	0.52790
	Case 5	-0.08047	0.46793	0.53207
	Case 6	-0.07002	0.47209	0.52791
	Case 7	-0.05975	0.47618	0.52382
	Case 8	-0.06993	0.47213	0.52787
	Case 9	-0.07087	0.47175	0.52825
	Case 10	-0.07006	0.47207	0.52793
Example 2	Case 1	0.05816	0.52319	0.47681
	Case 2	-0.00683	0.49728	0.50272
	Case 3	0.00798	0.50318	0.49682
	Case 4	-0.00682	0.49728	0.50272
	Case 5	0.01036	0.50413	0.49587
	Case 6	-0.00685	0.49727	0.50273
	Case 7	-0.01368	0.49454	0.50546
	Case 8	-0.00686	0.49727	0.50273
	Case 9	-0.00696	0.49722	0.50278
	Case 10	-0.00686	0.49727	0.50273

**Fig 10:**
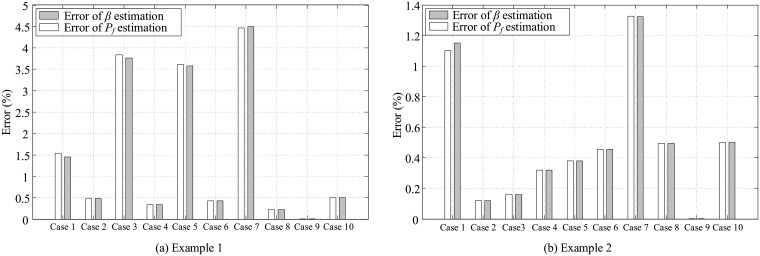
Relative difference between analytical and FFD results for (a) example 1 and (b) example 2.

Then, using Eqs (43) and (44), derivatives of *β* and *P*_*f*_ were calculated. The results are presented in the third and sixth columns of Table 5. In order to compare the results, the values obtained from the proposed algorithm for both *β* and *P*_*f*_ sensitivities were listed along with the values from the FFD (columns four and seven in Table 5.) Additionally, the relative error was estimated as follows:

$$\text{Error}=|\frac{\text{Analytical}-\text{FFD}}{\text{Analytical}}|\times 100,$$ (47)

where “Analytical” represents the results calculated by the proposed algorithm and “FFD” indicates the values obtained from forward finite difference method. The results are presented in the fifth and eights columns of Table 5. Moreover, the error values are plotted in Fig 10. Being adequately small, the error values indicate the accuracy of the proposed algorithm in the estimation of sensitivity derivatives.

**Table 5:** Comparison between the results obtained from analytical calculations and the FFD method.

Example	Case No.	*β* Sensitivity			*P*_*f*_ Sensitivity		
		FFD	Analytical	*β* Error (%)	FFD	Analytical	*P*_*f*_ Error (%)
Example 1	Case 1	−2.660 × 10^−2^	−2.699 × 10^−2^	1.456	1.058 × 10^−2^	1.074 × 10^−2^	1.537
	Case 2	1.523 × 10^−3^	1.531 × 10^−3^	0.482	−6.063 × 10^−4^	−6.092 × 10^−4^	0.481
	Case 3	−4.156 × 10^−4^	−4.318 × 10^−4^	3.762	1.653 × 10^−4^	1.718 × 10^−4^	3.839
	Case 4	9.831 × 10^−6^	9.797 × 10^−6^	0.341	−3.912 × 10^−6^	−3.899 × 10^−6^	0.342
	Case 5	−1.096 × 10^−3^	−1.136 × 10^−3^	3.580	4.359 × 10^−4^	4.522 × 10^−4^	3.617
	Case 6	1.817 × 10^−5^	1.809 × 10^−5^	0.422	−7.231 × 10^−6^	−7.200 × 10^−6^	0.422
	Case 7	2.062 × 10^−7^	2.159 × 10^−7^	4.502	−8.209 × 10^−8^	−8.593 × 10^−8^	4.469
	Case 8	6.547 × 10^−9^	6.532 × 10^−9^	0.223	−2.605 × 10^−9^	−2.599 × 10^−9^	0.224
	Case 9	−5.426 × 10^−3^	−5.426 × 10^−3^	0.000	2.159 × 10^−3^	2.159 × 10^−3^	0.003
	Case 10	1.451 × 10^−5^	1.444 × 10^−5^	0.498	−5.775 × 10^−6^	−5.746 × 10^−6^	0.498
Example 2	Case 1	6.502 × 10^−2^	6.428 × 10^−2^	1.151	−2.593 × 10^−2^	−2.564 × 10^−2^	1.102
	Case 2	2.829 × 10^−4^	2.832 × 10^−4^	0.120	−1.129 × 10^−4^	−1.130 × 10^−4^	0.120
	Case 3	1.484 × 10^2^	1.486 × 10^2^	0.159	−5.919 × 10^1^	−5.929 × 10^1^	0.158
	Case 4	7.546 × 10^−1^	7.571 × 10^−1^	0.320	−3.011 × 10^−1^	−3.020 × 10^−1^	0.320
	Case 5	1.434 × 10^10^	1.440 × 10^10^	0.379	−5.722 × 10^9^	−5.744 × 10^9^	0.378
	Case 6	1.713 × 10^7^	1.705 × 10^7^	0.455	−6.835 × 10^6^	−6.804 × 10^6^	0.455
	Case 7	−1.137 × 10^−2^	−1.152 × 10^−2^	1.324	4.536 × 10^−3^	4.597 × 10^−3^	1.327
	Case 8	5.490 × 10^−6^	5.463 × 10^−6^	0.493	−2.190 × 10^−6^	−2.179 × 10^−6^	0.493
	Case 9	−3.646 × 10^−4^	−3.646 × 10^−4^	0.000	1.455 × 10^−4^	1.455 × 10^−4^	0.000
	Case 10	1.374 × 10^−8^	1.367 × 10^−8^	0.500	−5.481 × 10^−9^	−5.454 × 10^−9^	0.500

## 6 Conclusion

In this paper, sensitivity analysis was performed for both the random and deterministic variables. For this purpose, after defining the performance function and the vector of involved variables, the problem was formulated and the required processes were established. The proposed algorithm was then implemented in Microsoft Excel using VBA programming language. Capability of the program for modeling and performing sensitivity analysis was then investigated. Next, two geotechnical and structural examples were presented to examine the proposed method. Finally, the proposed model was validated using the forward finite difference method. The main contributions of this paper are as follows:

- A step-by-step algorithm was presented to calculate the derivatives of safety index, *β*, and probability of failure, *P*_*f*_, with respect to the mean, standard deviation, and constant parameters.
- The proposed algorithm was programmed in Microsoft Excel environment, so that for any new model, the sensitivity analysis could be simply carried out without being engaged in any certain computational complexity.
- The proposed program was designed with no limitation on the number of parameters, ensuring that the program can cover models with virtually any number of variables.
- Sensitivity analysis of the deterministic variables was considered in the program. For this purpose, one should simply set the mean to the parameter value and set the standard deviation to zero.
- Formulation and problem solving implementation have been performed following the normalization of the input variables. That is, regardless of the type of distribution function governing the random variables, the normalized form of the variables is always input into the solving algorithm. Therefore, the proposed program could smoothly work for all the conventional distribution functions.

Last but not least, the proposed model is based on the AFOSM method. Thus, it has the same limitations as those suffered by the AFOSM method. For instance, the accuracy of the proposed method depends on non-linearity of the performance function. Hence, for highly non-linear functions, the results are associated with a margin of error. Moreover, for cases where an explicit form of the performance function is not available, the response surface methodology can be employed to provide the best-fit surface corresponding to the data instead of the performance function.

## Supporting information

S1 File Detailed data of Fig 7. (XLSX)

S2 File Microsoft Excel document and macros for reliability sensitivity analysis (RSA). (XLSM)
